# All-cause and cause-specific mortality differences between migrant workers and local workers: a population-based cohort study in Denmark

**DOI:** 10.1093/eurpub/ckaf058

**Published:** 2025-06-10

**Authors:** Karen Lau, George F Mkoma, Bertina Kreshpaj, Ligia Kiss, Cathy Zimmerman, Marie Norredam, Sally Hargreaves

**Affiliations:** Migrant Health Research Group, Institute for Infection and Immunity, City St. George’s, University of London and the Consortium for Migrant Worker Health, London, United Kingdom; Department of Global Health and Development, Faculty of Public Health and Policy, London School of Hygiene and Tropical Medicine, London, United Kingdom; Department of Epidemiology Research, Statens Serum Institut, Copenhagen, Denmark; Danish Research Centre for Migration, Ethnicity and Health, Section of Health Services Research, Department of Public Health, University of Copenhagen, Copenhagen, Denmark; Copenhagen Health Complexity Center, Department of Public Health, University of Copenhagen, Copenhagen, Denmark; Department of Global Health and Development, Faculty of Public Health and Policy, London School of Hygiene and Tropical Medicine, London, United Kingdom; Department of Global Health and Development, Faculty of Public Health and Policy, London School of Hygiene and Tropical Medicine, London, United Kingdom; Danish Research Centre for Migration, Ethnicity and Health, Section of Health Services Research, Department of Public Health, University of Copenhagen, Copenhagen, Denmark; Section of Immigrant Medicine, Department of Infectious Diseases, Hvidovre University Hospital, Hvidovre, Denmark; Migrant Health Research Group, Institute for Infection and Immunity, City St. George’s, University of London and the Consortium for Migrant Worker Health, London, United Kingdom

## Abstract

Migrants are prone to poor working conditions in high-risk industries, yet little is known about their mortality risk compared to local-born workers. This study compares all-cause and cause-specific mortality between foreign-born and local-born workers, and identifies at-risk foreign-born workers. A nationwide register-based cohort study was performed using data on migrant workers obtaining residence permits in Denmark during 2015–22. Comparison group comprised Danish-born workers matched by age and sex. Survival analysis using extended Cox model was used to estimate all-cause and cause-specific mortality. Subgroup analysis was conducted by region of birth, economic sector, and occupation. Male migrant workers from Central Europe, Eastern Europe, and Central Asia had higher risk of all-cause mortality than Danish-born workers (HR = 1.30 [95% CI: 1.09–1.54]), attributed to accident deaths (HR = 1.64 [1.06–2.53]), whereas migrants from other regions had lower risk. Migrant workers from these regions were more likely to work in high-risk economic sectors and occupations, such as agriculture and construction. When stratified by economic sector and by occupation, among the elementary occupations, migrant workers from these regions still had a higher risk of all-cause mortality (HR = 1.70 [1.10–2.64]) and accident mortality (HR = 1.51 [1.22–1.85]) than Danish-born workers. Migrant workers from Central Europe, Eastern Europe, and Central Asia are more likely to die from accidents than Danish-born workers. This increased risk was partially explained by their higher representation in at-risk sectors and occupations. There is a need to better understand the structural determinants of health faced by these migrants, particularly in elementary occupations, to prevent avoidable deaths.

## Introduction

Globally, the largest share of the 170 million international migrant workers is in Europe (32.2%) [1]. Recent events, including the COVID-19 pandemic, Qatar world cup and Paris Olympic games, have highlighted the health impacts of precarious or exploitative conditions in labour migrants [2]. Migrants are exposed to many occupational and environmental risks harmful to physical and mental health [3] and experience higher rates of fatal and non-fatal occupational injuries compared to local populations [4–6]. However, evidence underscores the heterogeneity among migrant workers, such as country of birth and economic sector, calling for better understanding of how migrant- and work-related factors interact to influence health outcomes [7, 8].

Denmark provides a suitable context to study labour migration and health, as its population-based registers offer longitudinal data on migrants’ employment and mortality [9]. The migrant population in Denmark continues to grow in the past 4 decades, reaching 15.9% of the population in 2024 who are immigrants or their descendants [10]. As Denmark’s integration strategy requires employment to be a condition for permanent residence since 2010 and for citizenship since 2016 [11], the Danish authorities have an obligation to ensure that migrants’ right to a safe and healthy work environment is protected. Despite Denmark’s strong labour protection laws, migrants continue to be prone to exploitative working conditions in high-risk jobs, such as trucking, construction, and agriculture [12].

While studies in Denmark have compared mortality between migrants and Danish-born, none has focused on migrant workers [13, 14]. Findings from Denmark could inform the wider European region given the steady increase in the share of migrants. Using Denmark’s population-based registers, this study aims to compare all-cause and cause-specific mortality risks between foreign-born and local-born workers, and to identify at-risk subgroups among foreign-born workers.

## Methods

### Study design and population

This is a nationwide register-based cohort study using Danish registries data. The cohort comprised migrants obtaining a residence permit in Denmark between 1 January 2015–31 December 2022. A Danish-born reference population was identified and matched 1:4 at an individual level by age and sex. The study population was restricted to participants aged 18 or above, with any history of economic activity during the study period.

### Data sources and linkage

We obtained data from the Danish Register of Causes of Death, the Ministry of Immigration and Integration, and Statistics Denmark [10, 15]. We linked datasets using the unique civil person registration number, which is assigned to each migrant in Denmark at date of residence and to each Danish-born at birth. Migrants who obtained more than one residence permit during the study period was counted only once based on their first permit obtained.

### Outcome variables

The primary outcomes were all-cause mortality and cause-specific mortality. Causes of death were coded according to the International Classification of Diseases, 10th revision (ICD-10).

### Exposure variables

The primary exposure was migrant status, defined as country of birth other than Denmark. Country of birth and region of birth, recoded referencing the Global Burden of Disease (GBD) regions [16], were analysed.

### Covariates

Covariates included sex, employment status, age at study entry, residence permit, economic sector, and occupation. Economic sector was recoded using Denmark's version of the European Union's Statistical Classification of Economic Activities in the European Community nomenclature, while occupation was recoded using Denmark’s version of the International Standard Classification of Occupations. These two Danish classifications are comparable with international standard classifications of economic activities and occupations.

### Statistical analyses

Survival analysis using the extended Cox model was used to estimate age-adjusted mortality rates, crude hazard ratios (HR), and adjusted HRs, reported with 95% confidence intervals. Age was used as follow-up time in Cox models to adjust for age effects on mortality. Sex, age at study entry, country of birth, and residence permit were analysed as time-fixed variables. Employment status, economic sector, and occupation were analysed as time-dependent variables. Start date was defined as date of residence permit for migrants, and that of the matched migrant for Danish-born. End date was defined as date of death, last emigration date, or study end date, whichever was earliest. Variables that did not violate the Cox proportional hazards assumption were retained in multiple Cox regression analysis, while those that did were analysed using stratification. Sub-group analysis was conducted for age, sex, region of birth and residence permit. R statistical software (version 4.3.2) was used to run all analyses.

### Ethics approval

This study obtained ethical approval through the University of Copenhagen (reference number 708907). We have adhered to Statistics Denmark’s data confidentiality policy, and informed consent of individual study participants was not needed. No further approval is required with regard to register-based research according to the Danish law.

## Results

### Study population

The study population comprised 119 370 migrant workers and 795 763 Danish-born workers (Table 1). Migrant workers were more likely to be male and younger than Danish workers, while female migrant workers were younger than male migrant workers. The majority (66.6%) of migrant workers held EU/EEA residence permits, and over half (51.3%) were born in Central Europe, Eastern Europe and Central Asia. The top ten countries of birth were Romania, Poland, Ukraine, India, Lithuania, Bulgaria, Italy, United Kingdom, Germany, and Argentina.

**Table 1.** Characteristics of study population

		All workers				Male workers				Female workers			
		All migrant workers		All Danish workers		Male migrant workers		Male Danish workers		Female migrant workers		Female Danish workers	
*N*		119 370		795 763		83 468		504 950		35 902		290 813	
Sex	Male	83 468	69.9%	504950	63.5%	–	–	–	–	–	–	–	–
	Female	35 902	30.1%	290813	36.5%	–	–	–	–	–	–	–	–
Age at study entry (mean)		31.9		33.2		32.5	–	33.6	–	30.4	–	32.7	–
Permit type^a^	EU/EEA	79 479	66.6%	–		57 131	68.4%	–	–	22 348	62.2%	–	–
	Work	28 382	23.8%	–		20 100	24.1%	–	–	8282	23.1%	–	–
	Refugees or asylum seekers	226	0.2%	–		164	0.2%	–	–	62	0.2%	–	–
	Family reunification	2856	2.4%	–		903	1.1%	–	–	1953	5.4%	–	–
	Study	8640	7.2%	–		5283	6.3%	–	–	3357	9.4%	–	–
	Others	76	0.1%	–		34	0.0%	–	–	42	0.1%	–	–
Region of birth	Western Europe and North America	27 174	22.8%	–		18 304	21.9%	–	–	8870	24.7%	–	–
	Central Europe, Eastern Europe, and Central Asia	61 219	51.3%	–		44 865	53.8%	–	–	16 354	45.6%	–	–
	Latin America and Caribbean	7766	6.5%	–		4611	5.5%	–	–	3155	8.8%	–	–
	Southeast Asia, East Asia, and Oceania	8078	6.8%	–		4352	5.2%	–	–	3726	10.4%	–	–
	South Asia	9509	8.0%	–		7410	8.9%	–	–	2099	5.8%	–	–
	Sub-Saharan Africa, North Africa and Middle East	5550	4.6%	–		3872	4.6%	–	–	1678	4.7%	–	–
	Others	29	0.0%	–		24	0.0%	–	–	5	0.0%	–	–
	Missing	45	0.0%	–		30	0.0%	–	–	15	0.0%	–	–
Country of birth (top 10)	Romania	17 007	14.2%	–		13 033	15.6%	–		3974	11.1%	–	
	Poland	15 401	12.9%	–		11 977	14.3%	–		3424	9.5%	–	
	Ukraine	8948	7.5%	–		6355	7.6%	–		2593	7.2%	–	
	India	7180	6.0%	–		5653	6.8%	–		1527	4.3%	–	
	Lithuania	6345	5.3%	–		4340	5.2%	–		2005	5.6%	–	
	Bulgaria	4366	3.7%	–		3128	3.7%	–		1238	3.4%	–	
	Italy	4241	3.6%	–		3114	3.7%	–		1127	3.1%	–	
	United Kingdom	4237	3.5%	–		3161	3.8%	–		1076	3.0%	–	
	Germany	4111	3.4%	–		2425	2.9%	–		1686	4.7%	–	
	Argentina	4100	3.4%	–		2370	2.8%	–		1730	4.8%	–	

a Sum total of permit types may exceed the total number of study participants, because some study participants may have obtained more than one residence permit during the study period.

### All-cause mortality

193 migrant worker deaths were recorded among 470 462 person-years, while 1570 Danish worker deaths were recorded among 2 902 768 person-years (Table S1). The age-adjusted all-cause mortality rate in migrant workers (41.0 [95% CI: 35.2–46.8] per 100 000 person-years) was lower than that in Danish workers (54.1 [51.4–56.8] per 100 000 person-years). In both migrant and Danish workers, all-cause mortality rate of males was more than two times higher than that of females, and all-cause mortality rate of being in self-employed or other employment status was more than three times higher than that of being employed.

After adjusting for sex, age, and employment status, the risk of all-cause mortality between migrant workers and Danish workers did not differ when migrants were analysed as a single population (HR = 0.89 [0.76–1.03]), but differed when analysed by region of birth. Migrant workers from Central Europe, Eastern Europe and Central Asia had a higher risk of all-cause mortality than Danish workers (HR = 1.30 [1.09–1.54]), while those from most other regions had a lower risk (Table 2). This mortality pattern was only observed among males. Among male workers, the adjusted HR for all-cause mortality in migrants from Central Europe, Eastern Europe and Central Asia was 1.35 [1.12–1.62], while migrants from other regions was 0.44 [0.32–0.61], compared to Danish-born. Among female workers, there was no difference in all-cause mortality rate between migrants from Central Europe, Eastern Europe and Central Asia (HR = 1.03 [0.66–1.62]), or other regions (HR = 0.55 [0.30–1.01]) and the Danish-born.

**Table 2.** Adjusted hazard ratio of all-cause mortality in migrant workers compared to Danish workers, by region of birth, 2015–22

	Death	Person-years	Rate^a^	95% CI	Crude HR	95% CI	Adjusted HR^b^	95% CI
Danish	1570	2 902 768	54.1	(51.4–56.8)	1		1	
All migrants	193	470 462	41.0	(35.2–46.8)	0.85	(0.73–0.98)	0.89	(0.76–1.03)
*By region of birth*								
Western Europe and North America	30	97 668	30.7	(19.7–41.7)	0.54	(0.37–0.77)	0.58	(0.41–0.84)
Central Europe, Eastern Europe, and Central Asia	144	236 599	60.9	(50.9–70.8)	1.27	(1.07–1.51)	1.30	(1.09–1.54)
Latin America and Caribbean	3	26 132	11.5	(0.0–24.5)	0.31	(0.10–0.97)	0.32	(0.11–1.01)
Southeast Asia, East Asia, and Oceania	6	37 818	15.9	(3.2–28.6)	0.37	(0.17–0.82)	0.42	(0.19–0.94)
South Asia	5	42 043	11.9	(1.5–22.3)	0.29	(0.12–0.70)	0.31	(0.13–0.74)
Sub-Saharan Africa, North Africa and Middle East	5	29 877	16.7	(2.1–31.4)	0.32	(0.13–0.77)	0.32	(0.13–0.76)

a Age-adjusted rate per 100 000 person-years.

b Adjusted for sex, age, and employment status.

### Cause-specific mortality

**Figure 1.**
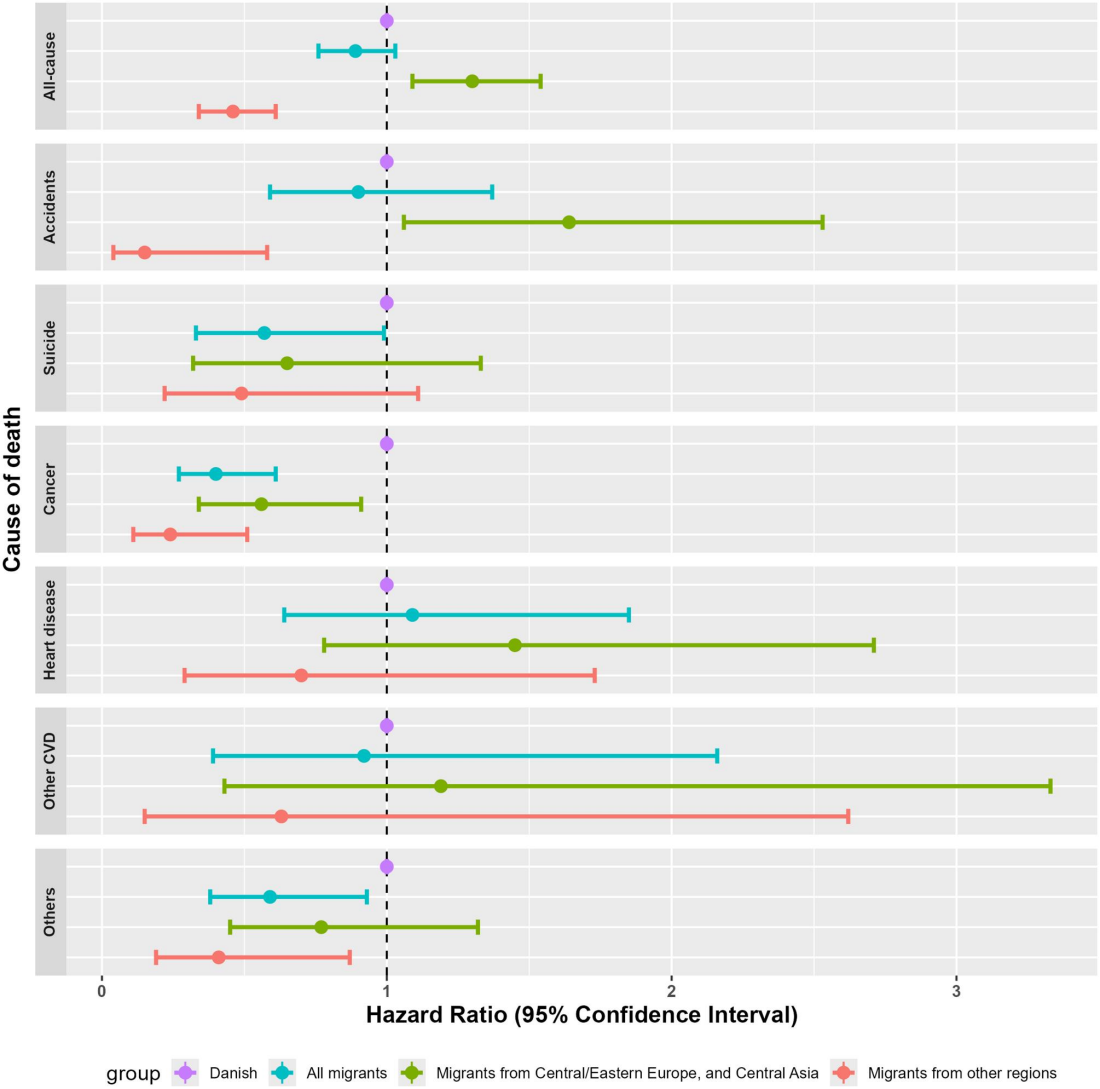
Adjusted hazard ratio* of all-cause mortality and top five cause-specific mortality in migrant workers compared to Danish workers, by region of birth, 2015-2022 for all-cause mortality, 2015–21 for cause-specific mortality. *Adjusted for sex, age, and employment status.

The most common causes of death among migrant workers were cancer (25%), accidents (23%), heart diseases (15%), suicide (13%), and other cardiovascular diseases (6%), while for Danish workers were cancer (35%), accidents (16%), suicide (14%), heart diseases (9%), and other cardiovascular diseases (4%). When taking into account population at-risk, the only cause of death in which migrant workers had a higher risk of mortality than Danish workers was accident deaths (Fig. 1). While migrant workers on the whole did not differ from Danish workers on risk of accident deaths (HR = 0.90 [0.59–1.37]), a different picture emerged when analysed by region of birth. Migrant workers from Central Europe, Eastern Europe and Central Asia had a higher risk of accident deaths than Danish workers (HR = 1.64 [1.06–2.53]), while migrants from other regions had a lower risk (HR = 0.15 [0.04–0.58]). Sub-group analysis by region of birth across all causes of death showed that migrant workers from Central Europe, Eastern Europe and Central Asia was the only group that had a higher mortality risk than Danish workers, exhibited through their higher risk of accident deaths. While only half of migrant workers in this study were from these regions, almost all (92%) of the accident deaths among migrant workers occurred within this population.

When analysed by ICD-10 codes, more than one third (39%) of the accident deaths in migrant workers from Central Europe, Eastern Europe and Central Asia were transport accidents, such as vehicle collision and fishing boat accident. The remaining accident deaths were other external causes of accidental injury, such as fall, struck by falling object, and exposure to fire. When accident deaths were analysed by place of occurrence, the proportion of workers who died in production and workshop areas was three times higher among migrant workers from these regions than that in Danish-born workers.

### Economic sector and occupation

Migrants were more likely to work in higher risk sectors and occupations than Danish workers, including agriculture, forestry, and fishing sector (13.0% vs 1.3%) and in elementary occupations (12.3% vs 5.1%) (Table S2). Elementary occupations are those that involve the performance of simple and routine tasks which may require the use of hand-held tools and considerable physical effort, such as cleaners, agricultural or construction labourers, transport or freight handlers, and package deliverers. In particular, migrants from Central Europe, Eastern Europe, and Central Asia were over-represented in the agriculture, forestry and fishing (24.7%) and construction (11.9%) sectors, and in the elementary occupations (17.8%), compared to both Danish workers and migrants from other regions. When analysed by sex, male migrant workers were more likely to work in agriculture, forestry and fishing (14.5% vs 9.8%), construction (10.4% vs 1.1%), and manufacturing, mining and quarrying (13.3% vs 9.6%) than female migrant workers, and less likely to work in public administration, education and health (8.4% vs 15.6%) and other business services (14.6% vs 22.2%).

**Figure 2.**
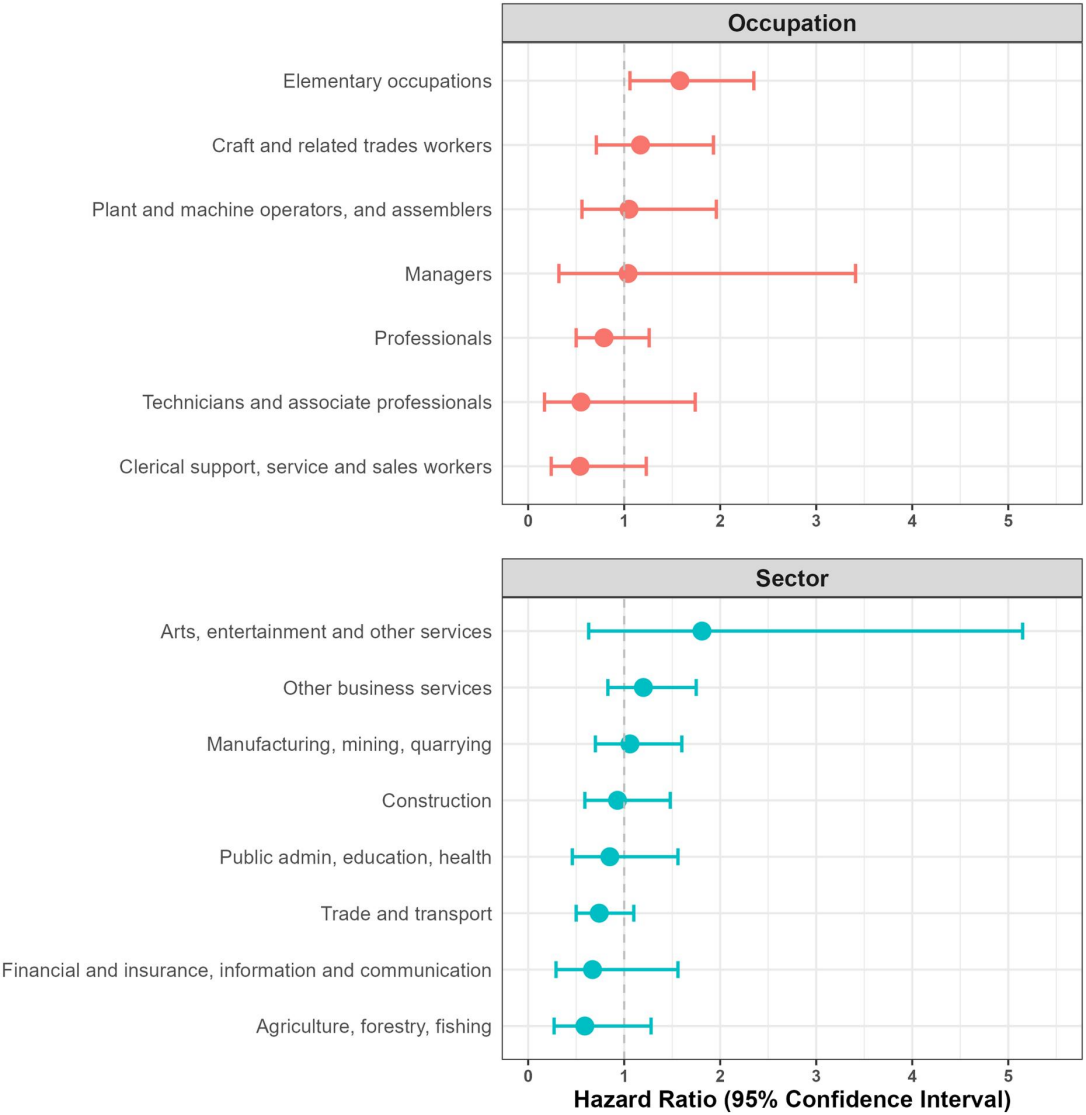
Adjusted hazard ratio* of all-cause mortality in migrant workers compared to Danish workers, stratified by economic sector and by occupation. *Adjusted for sex, age, and employment status.

When stratified by sector and by occupation, the risk of all-cause mortality between migrant workers and Danish workers did not differ, except in the elementary occupations (Fig. 2). Among the elementary occupations, migrants had a higher risk of all-cause mortality than Danish workers (HR = 1.58 [1.06–2.35]). When analysed by region of birth among elementary occupations, migrant workers from Central Europe, Eastern Europe and Central Asia again had a higher risk of mortality than Danish workers (HR = 1.70 [1.10–2.64]), explained by their higher risk of accident deaths (HR = 1.51 [1.22–1.85]), of which the most common occupations among these migrant worker accident deaths were warehouse and construction work, whereas migrants from other regions did not have a higher risk of mortality.

## Discussion

This nationwide register-based cohort study found that the mortality pattern of migrant workers differed by countries of birth. The only group of migrant workers who experienced worse mortality outcomes than Danish-born workers were migrant workers from Central Europe, Eastern Europe and Central Asia, with higher risk of all-cause mortality explained by accident deaths. Migrant workers from other regions had lower all-cause and accident mortality risks than Danish-born workers. Previous studies in Denmark found that migrants holding refugee or family reunification residence permits have lower all-cause and accident mortality compared with Danish-born [13, 14], while migrant workers who recently arrived Denmark are at increased risk of work injuries [17, 18]. This study adds to this body of literature by identifying a health disparity between migrant workers and local workers manifested as higher risk of accident deaths among some migrant worker groups. Informed by social epidemiologic theories [19], we explore how broader structural determinants of health interact with individual factors to explain the higher risk of accident deaths in this largest migrant worker population in Denmark.

A rather alarming finding is that migrant workers from Central Europe, Eastern Europe and Central Asia bore a disproportionately high burden of accident deaths among all migrant workers, despite only comprising half of this population. Among the accident deaths that occurred in these migrant workers, more than a third were transport-related deaths. Although we were not able to differentiate whether the accident deaths were occupational-related, the latest 2021 GBD study identified occupational injuries as the leading risk factor for transport injuries in Denmark [20]. In Denmark, as is globally, there is a rising trend of workers in the platform economy, particularly those referred to by the ILO as ‘location-based platforms’, which are platforms mediating geographically tethered tasks such as ride hailing and food delivery. The majority of platform workers in Denmark are migrants, such as Wolt couriers, and is often their first or one of their first jobs in Denmark [21]. Working in the platform economy is associated with increased risk of work-related accidents, due to working at a rapid pace without breaks, lack of appropriate training, and working in transport-related jobs at risk of road accidents [22, 23]. There is also evidence that the psychosocial hazards associated with working in the platform economy may increase the risk of cardiovascular disease and mortality in these workers [24].

In addition, fatigue and sleepiness are also known risk factors for increased risk of accidents, especially traffic accidents [25, 26]. Workers who work night shifts or long hours become more tired and less alert, causing them to be more accident-prone during and after work [27]. Polish workers in Denmark were found to be more than twice as likely to work night shifts than Danish workers, such as newspaper delivery, warehouse work, and non-domestic cleaning, thus increasing their risk of accidents [28].

Labour segmentation is another explanation for the increased risk of accident deaths observed in certain migrant worker populations. Migrant workers from Central Europe, Eastern Europe and Central Asia were disproportionately represented in labour-intensive sectors and occupations (such as agriculture, construction, and elementary occupations) compared with Danish workers and migrant workers from other regions. These jobs are known to have higher rates of occupational injuries due to the nature of work tasks, such as operating heavy machinery, performing repetitive motions, and risk of falling [29]. Some characteristics of these sectors also make migrants particularly vulnerable to work injuries, such as the construction industry’s widespread use of subcontracting or hiring migrants without a contract and practice of holding safety meetings in the Danish language [30]. Stratification analysis found that sector and occupation only partially explained the mortality difference observed, and that migrants working in the elementary occupations experienced additional risks beyond sector and occupation that exposed them to higher risks of accident deaths. Representation and enforcement gaps in elementary occupations highly represented by migrants may explain their inadequate safety protection in Denmark [31]. Shop steward representation and collective agreements coverage are lower among migrants in Denmark, contributing to unsafe work environments as reported among Polish workers [28]. Enforcement gaps are also common, as migrants face language barriers, have less knowledge about the Danish labour regulations, and tend to avoid reporting workplace violations for fear of reprisal [31, 32]. Power imbalance between migrants and their employers was found to influence occupational safety protection [32], which could also explain why migrants in the elementary occupations were at higher risk of accident deaths in contrast to those in the professional occupations. Labour segmentation along the gender dimension could also explain the higher risk of mortality in male migrant workers compared to female migrant workers, as our findings showed that male migrants were more likely to be in labour-intensive sectors, while female migrants were more likely to be in office- or administrative-based sectors.

On top of labour segmentation by industry or occupation, work tasks within the same industry or occupation can also be highly segmented. Migrant workers from Poland and Romania were found to face greater work pressures than Danish workers in the same workplace, including expectation to complete the same task faster and being assigned more dangerous tasks, such as lifting heavier loads, given outdoor tasks on a rainy day, and operating cranes during strong winds [30]. Among migrant workers, work tasks are also segmented by nationality, with Romanians and Bulgarians taking up the lowest paid jobs with the most strenuous working conditions [31].

Health care access could be another explanation for the observed mortality difference. Despite Denmark’s tax-funded health care system where everyone with the right to residency are eligible for free health care, the introduction of a fee for health care interpretation since 2018 was found to reduce utilization of interpretation services as well as health care services among migrants [33, 34], which would affect migrant workers with lower wages the most such as those in the elementary occupations.

The key strength of this study is using nationwide longitudinal linked registers, which enables complete and objective ascertainment of employment exposure and death status of all migrant workers who obtained residence permit in Denmark. This study has several key limitations. First, only migrant workers who obtained residence permit were included, thereby excluding those undocumented or on temporary residence, who are more likely to work in unsafe conditions, thus our mortality risk estimates are likely underestimated. A Swedish study has found that undocumented migrants were more likely to die from external causes than locals, but these death certificates were not recorded in the Swedish Cause of Death Register [35]. Second, although our study population was restricted to workers, we do not have information on whether specific causes of death were occupational-related. On the other hand, Denmark does not include traffic accidents commuting to or from work in its definition of occupational accidents [36], whereas our study was able to capture these work-related traffic accident deaths not reflected in the official statistics. Third, our gender-based findings should be interpreted as exploratory, as the absence of statistical difference may be due to small sample size and hence insufficient power to detect true difference. Fourth, our study only had access to limited data regarding the working conditions and employment terms [37] of study participants. For instance, low income and long working hours were explored as covariates, but missing data prevented us from including these in subsequent analysis. Fifth, we were not able to include other key social determinants of health, such as education and vocational training, as potential confounders in our analysis. This is due to the complexity of comparing migrants’ qualifications before and after arriving Denmark with that of the Danish-born, which could be explored in future studies. Sixth, misclassification of specific causes of death in the registry data is possible since only 5% of deaths in Denmark are examined by autopsy [38], which may be more pronounced in migrants due to incomplete medical history and language barriers with families. Finally, there may be a risk of selection bias where migrant workers who become ill may return to their country of origin, although this is unlikely in Denmark [39].

This study exposes the need to prevent avoidable accident deaths among migrant workers, and points to interventions that shift towards the structural determinants of health [40]. In view of findings on labour segmentation by migrant status, efforts should be targeted to improve the occupational safety and health of sectors and occupations with highest risk of occupational injuries, including agriculture, construction, and elementary occupations. Sector-specific prevention approaches have the potential to reduce health inequities between migrant workers and Danish-workers since these sectors are disproportionately represented by migrants, as well as prevent work injuries in general among all workers in these sectors, thereby benefiting both migrants and Danish-born alike. For instance, in Austria, where the majority of live-in care workers are migrants, the law requires that live-in care workers be provided with accommodation that is compliant with health, construction, and fire regulations. To counter the power imbalance between migrants and their employers, state and non-state actors should address not only individual-level risk factors (such as migrants’ lower understanding of their workplace rights), but also structural factors (such as inadequate labour protection through collective agreements). For instance, Italy grants ‘special cases’ residence permits to migrant workers in irregular situations in an exploitative situation to enable migrant workers to cooperate with the inspectorate without fearing negative repercussions. Placed-based interventions could be implemented by outreach to worksites where migrants tend to gather to deliver language- and culture-sensitive messages. For instance, the Swedish and Finnish authorities have produced multilingual guidebooks for agricultural workers. Unions and worker organizations should prioritize strengthening migrant worker representation and labour inspection in workplaces highly represented by migrant workers, particularly the elementary occupations, which helps to hold employers accountable for their occupational health and safety responsibilities. While labour inspectors in Denmark are not required to perform other duties other than those related to workers’ safety and health, it is important to ensure that migrant workers in irregular situations are granted their due rights in practice. Given the rising trend of migrants working in the platform economy, labour regulations must be able to respond to these newer forms of work to enforce occupational safety standards. As Denmark’s integration policy requires that employment is a condition to apply for permanent residence and citizenship, the authorities have a moral obligation to protect migrants at work and to ensure that labour protection laws effectively cover migrant workers. The bilateral agreements signed between Denmark and various migrant-sending countries, including Poland, Lithuania, Latvia, Slovakia, and most recently Romania, are welcome measures to monitor labour violations of employers hiring migrants in Denmark, although specific provisions and measures regarding occupational safety and health prevention will still be necessary to protect migrant workers from getting injured in the first place. Beyond Denmark, findings may also be relevant to other high-income countries with increasing migrant populations, particularly where work requirement is specified in integration policies or is a condition to qualify for social protection benefits.

## Supplementary Material

ckaf058_Supplementary_Data

## Data Availability

Data can be accessed through Statistics Denmark by acquiring the necessary researcher agreements. Key points Migrant workers from Central Europe, Eastern Europe and Central Asia, who comprise the largest share of migrants in Denmark, were the only migrant group that had higher all-cause mortality and accident mortality compared to Danish-born workers. These migrant workers bore a disproportionately high burden (92%) of accident deaths among all migrant workers despite only comprising around half of the migrant worker population. Labour segmentation by country of birth into higher risk sectors and occupations partly explained the higher risk of accident deaths in these migrant workers. Among workers in the elementary occupations, migrants experienced additional risk factors beyond sector and occupation that exposed them to higher risks of accident deaths compared to Danish-born workers. Interventions and policies must address not only individual-level risk factors (such as enhancing migrant workers’ awareness of their workplace rights), but also structural level power imbalances (such as strengthening labour protection for migrants through collective agreements and labour laws).
