# Supplementary material for: All-cause and cause-specific mortality differences between migrant workers and local workers: a population-based cohort study in Denmark
Source: Eur J Public Health. 2025 Jun 10;35(4):672–9. doi: 10.1093/eurpub/ckaf058 (PMC12311336; doi:10.1093/eurpub/ckaf058)
Supplement: ckaf058_Supplementary_Data [file ckaf058_supplementary_data.docx]

**Supplementary Material**

**All-cause and cause-specific mortality differences between migrant workers and local workers: a population-based cohort study in Denmark**

Karen Lau, MSc, George F Mkoma, PhD, Bertina Kreshpaj, PhD, Ligia Kiss, PhD, Cathy Zimmerman, PhD*, Marie Norredam, PhD*, Sally Hargreaves, PhD* (*Joint senior authors)

Table S1. All-cause mortality rates of migrant workers and local workers in Denmark, 2015-2022.

|  | | All migrant workers (n = 119370) | | | | All Danish workers (n = 795763) | | | |
| --- | --- | --- | --- | --- | --- | --- | --- | --- | --- |
|  |  | **Death** | **Person-years** | **Rate*** | **95%CI** | **Death** | **Person-years** | **Rate*** | **95%CI** |
| Total | | 193 | 470462 | 41.0 | (35.2 – 46.8) | 1570 | 2902768 | 54.1 | (51.4 – 56.8) |
| Sex | Male | 161 | 318859 | 50.5 | (42.7 – 58.3) | 1301 | 1964864 | 66.2 | (62.6 – 69.8) |
|  | Female | 32 | 151602 | 21.1 | (13.8 – 28.4) | 269 | 937904 | 28.7 | (25.3 – 32.1) |
| Age at study entry | 18-25 | 10 | 102542 | 9.8 | (3.7-15.8) | 132 | 615375 | 21.5 | (17.8-25.1) |
|  | 26-35 | 53 | 243722 | 21.7 | (15.9-27.6) | 479 | 1436744 | 33.3 | (30.4-36.3) |
|  | 36-45 | 58 | 87162 | 66.5 | (49.4-83.7) | 366 | 569671 | 64.2 | (57.7-70.8) |
|  | 46-55 | 43 | 30254 | 142.0 | (99.6-185.0) | 341 | 219779 | 155.0 | (139.0-172.0) |
|  | 56 and above | 29 | 6783 | 428.0 | (272.0-583.0) | 252 | 61198 | 412.0 | (361.0-463.0) |
| Employment status | Employed | 116 | 396040 | 29.3 | (24.0-34.6) | 865 | 2323799 | 37.2 | (34.7-39.7) |
|  | Self-employed or other | 77 | 74416 | 103.0 | (80.4-127.0) | 705 | 578871 | 122.0 | (113.0-131.0) |
| Sector | Agriculture, forestry, fishing | 14 | 61047 | 22.9 | (10.9-35.0) | 24 | 37610 | 63.8 | (38.3-89.3) |
|  | Construction | 21 | 34913 | 60.1 | (34.4-85.9) | 146 | 251578 | 58.0 | (48.6-67.4) |
|  | Manufacturing, mining, quarrying | 26 | 56836 | 45.7 | (28.2-63.3) | 187 | 321819 | 58.1 | (49.8-66.4) |
|  | Trade and transport | 28 | 98295 | 28.5 | (17.9-39.0) | 258 | 590045 | 43.7 | (38.4-49.1) |
|  | Financial and insurance, information and communication | 6 | 40050 | 15.0 | (3.0-27.0) | 62 | 237132 | 26.1 | (19.6-32.7) |
|  | Other business services | 35 | 80403 | 43.5 | (29.1-58.0) | 145 | 355874 | 40.7 | (34.1-47.4) |
|  | Public admin, education, health | 11 | 50375 | 21.8 | (8.9-34.7) | 235 | 799375 | 29.4 | (25.6-33.2) |
|  | Arts, entertainment and other services | 4 | 9172 | 43.6 | (0.9-86.3) | 31 | 101074 | 30.7 | (19.9-41.5) |
|  | Not stated | 48 | 39346 | 122.0 | (87.5-157) | 482 | 207531 | 232.0 | (212-253) |
| Occupation | Managers | 3 | 7874 | 38.1 | (0.0-81.2) | 36 | 89913 | 40.0 | (27.0-53.1) |
|  | Professionals | 20 | 116702 | 17.1 | (9.6-24.6) | 214 | 829360 | 25.8 | (22.3-29.3) |
|  | Technicians and associate professionals | 3 | 20876 | 14.4 | (0.0-30.6) | 105 | 295673 | 35.5 | (28.7-42.3) |
|  | Clerical support, service and sales workers | 6 | 35951 | 16.7 | (3.3-30.0) | 203 | 594646 | 34.1 | (29.4-38.8) |
|  | Skilled agricultural, forestry and fishery workers | 0 | 1671 | 0.0 | 0 | 10 | 25399 | 39.4 | (15.0-63.8) |
|  | Craft and related trades workers | 17 | 25064 | 67.8 | (35.6-100.0) | 166 | 313918 | 52.9 | (44.8-60.9) |
|  | Plant and machine operators, and assemblers | 11 | 14083 | 78.1 | (31.9-124.0) | 108 | 111413 | 96.9 | (78.7-115) |
|  | Elementary occupations | 38 | 57914 | 65.6 | (44.8-86.5) | 75 | 148659 | 50.5 | (39.0-61.9) |
|  | Armed forces occupations | 0 | 9 | 0.0 | 0 | 7 | 30959 | 22.6 | (5.9-39.4) |
|  | Not informed | 95 | 190313 | 49.9 | (39.9-60.0) | 646 | 462731 | 140.0 | (129.0-150.0) |

*Age-adjusted rate per 100,000 person-years

Table S2. Distribution of migrant workers and Danish workers across sectors and occupations, in person-years.

|  | | All Danish workers | | All migrant workers | | Central Europe, Eastern Europe, and Central Asia | | Other regions | |
| --- | --- | --- | --- | --- | --- | --- | --- | --- | --- |
| Sector | Agriculture, forestry, fishing | 37610 | 1.3% | 61047 | 13.0% | 58442 | 24.7% | 2604 | 1.1% |
|  | Construction | 251578 | 8.7% | 34913 | 7.4% | 28091 | 11.9% | 6817 | 2.9% |
|  | Manufacturing, mining, quarrying | 321819 | 11.1% | 56836 | 12.1% | 28150 | 11.9% | 28651 | 12.3% |
|  | Trade and transport | 590045 | 20.3% | 98295 | 20.9% | 44386 | 18.8% | 53860 | 23.1% |
|  | Financial and insurance, information and communication | 237132 | 8.2% | 40050 | 8.5% | 7898 | 3.3% | 32112 | 13.7% |
|  | Other business services | 355874 | 12.3% | 80403 | 17.1% | 42032 | 17.8% | 38304 | 16.4% |
|  | Public admin, education, health | 799375 | 27.5% | 50375 | 10.7% | 7034 | 3.0% | 43326 | 18.5% |
|  | Arts, entertainment and other services | 101074 | 3.5% | 9172 | 1.9% | 2727 | 1.2% | 6440 | 2.8% |
|  | Not stated | 207531 | 7.1% | 39346 | 8.4% | 17829 | 7.5% | 21500 | 9.2% |
| Occupation | Managers | 89913 | 3.1% | 7874 | 1.7% | 1077 | 0.5% | 6783 | 2.9% |
|  | Professionals | 829360 | 28.6% | 116702 | 24.8% | 16841 | 7.1% | 99820 | 42.7% |
|  | Technicians and associate professionals | 295673 | 10.2% | 20876 | 4.4% | 5669 | 2.4% | 15177 | 6.5% |
|  | Clerical support, service and sales workers | 594646 | 20.5% | 35951 | 7.6% | 12481 | 5.3% | 23440 | 10.0% |
|  | Skilled agricultural, forestry and fishery workers | 25399 | 0.9% | 1671 | 0.4% | 1395 | 0.6% | 276 | 0.1% |
|  | Craft and related trades workers | 313918 | 10.8% | 25064 | 5.3% | 21459 | 9.1% | 3573 | 1.5% |
|  | Plant and machine operators, and assemblers | 111413 | 3.8% | 14083 | 3.0% | 12555 | 5.3% | 1513 | 0.6% |
|  | Elementary occupations | 148659 | 5.1% | 57914 | 12.3% | 42227 | 17.8% | 15676 | 6.7% |
|  | Armed forces occupations | 30959 | 1.1% | 9 | 0.0% | 3 | 0.0% | 5 | 0.0% |
|  | Not informed | 462731 | 15.9% | 190313 | 40.5% | 122889 | 51.9% | 67362 | 28.8% |
